# Biotic Interaction Underpins the Assembly Processes of the Bacterial Community Across the Sediment–Water Interface in a Subalpine Lake

**DOI:** 10.3390/microorganisms12122418

**Published:** 2024-11-25

**Authors:** Xue Wang, Jinxian Liu, Jiali Ren, Baofeng Chai

**Affiliations:** 1 Institute of Loess Plateau, Shanxi University, Taiyuan 030006, China; 202113202005@email.sxu.edu.cn (X.W.); 202323202021@email.sxu.edu.cn (J.R.); bfchai@sxu.edu.cn (B.C.); 2 Shanxi Key Laboratory of Ecological Restoration for Loess Plateau, Shanxi University, Taiyuan 030006, China

**Keywords:** assembly process, biotic interaction, protozoan, important taxa, sediment–water interface

## Abstract

The sediment–water interface is the most active region for biogeochemical processes and biological communities in aquatic ecosystems. As the main drivers of biogeochemical cycles, the assembly mechanisms and the distribution characteristics of microbial communities at this boundary remain unclear. This study investigated the microbial communities across the sediment–water interface in a natural subalpine lake in China. The results indicated that the diversity of bacterial communities in middle sediment was significantly higher than that in overlying water and other sediments (*p* < 0.001). Pearson’s correlation analysis indicated that the diversity was significantly influenced by biotic factors (e.g., diversity of fungus, protozoan and alga) and physicochemical parameters (e.g., total carbon, total organic carbon, nitrate, ammonium and pH) (*p* < 0.01). Null model analysis revealed that the homogeneous selection dominated the assembly of the bacteria community in sediment, whereas the heterogeneous selection dominated that in overlying water. The least squares path analysis showed that interactions between protozoa and bacteria had a greater impact on bacterial community assembly (*p* < 0.001). Important taxa influence the assembly by regulating biotic interactions. These findings provided a basis for understanding the importance of biotic interactions in maintaining subalpine lakes’ ecosystems across the sediment–water interface.

## 1. Introduction

The sediment–water interface is an environmental boundary with significant differences in physicochemical and biological characteristics and is the most active area for biogeochemical processes in aquatic ecosystems [1,2,3]. Microbial communities play an important role in driving many biogeochemical processes [3], e.g., oxidation and reduction [4], adsorption and desorption [5], migration and transformation [6], etc. As a core issue in ecology [7,8], the assembly mechanism of the microbial community in this sediment–water interface is still unclear.

Ecological deterministic and stochastic processes jointly govern the community assembly, and the relative roles of these are different for communities in different habitats [9,10]. Dai et al. proposed the “Hunger Games” hypothesis [11] on which the relative importance of deterministic and stochastic processes in the assembly of microbial communities might vary depending on biotic interactions and environmental conditions. Abiotic physicochemical properties and biotic interaction among organisms are two aspects of deterministic process driving the assembly processes of various communities in many habitats [12]. The environmental heterogeneity on the interface cross-habitat creates niche opportunities for microorganisms [13]. This environmental heterogeneity drives the assembly of microbial communities, which indicate that the deterministic process plays an important role in this process [14]. In recent years, the effects of abiotic environmental factors on the assembly processes of microbial communities have been intensively studied; meanwhile, the impact of biotic interactions on this process is receiving increasing attention [12,15].

In lakes, algae, protozoa, fungi, and bacteria are primary producers, bacterivores and decomposers, respectively [16,17]. These microorganisms form complex biotic interactions (such as predation, symbiosis and parasitism) through material cycling, energy flow and information exchange, thereby maintaining the stability of the structure of the lake ecosystem [18,19]. For example, the predation pressure of protozoa significantly impacts the composition of bacteria and fungi. As the key food source of protozoa, the abundance of bacteria and fungi may in turn affect their distribution [20]. However, how biotic interactions and environmental factors synergistically regulate the structure and assembly of bacterial communities in the sediment–water interface with high environmental heterogeneity remains unknown.

The multi-network analysis method can infer the microbial interactions in a community. In this method, positive and negative edges in the network could reflect the potential cooperative and competitive relationships in communities [21]. The positive-to-negative edges ratio (P/N) can represent biotic interactions and impact factors on community stability [22]. In addition, taxa are also important indicators reflecting biotic interactions and ecological assembly processes. In different habitats, whether taxa have a higher or lower abundance in microbial communities has an important impact on community structure and stability [23]. Random forests analysis methods eliminate the influence of the abundance of taxonomic groups and could identify the real important taxa for the habitat in rank order of their contribution to the predictive accuracy of the mode [24]. Through analyzing biotic interactions and important taxa, it can indicate the regulation mechanism of biotic interactions regarding the assembly of bacterial communities in the sediment–water interface.

Despite extensive studies on bacterial, fungal and protozoan communities in the water of subalpine lakes [18,20,25], the community assembly and biotic interaction of microbial communities in the sediment–water interface remain relatively unexplored. In this study, we collected overlying water and sediment samples at different depths in a subalpine lake, evaluated the diversity of prokaryotic and eukaryotic microorganisms and revealed the assembly processes of the bacterial community. The following hypotheses were proposed: (1) environmental heterogeneity is the main driver of the diversity of the bacterial community in cross-habitat ecosystems; (2) protozoa have a greater impact on the diversity of the bacterial community across the sediment–water interface; (3) biotic interactions have a significant impact on the assembly of bacterial communities, and important taxa play a crucial role in this process. It is great significance for understanding the maintenance mechanism of bacterial community diversity in the sediment–water interface.

## 2. Materials and Methods

### 2.1. Sampling and Physicochemical Properties

Six duplicate samples of sediment cores were collected from the Gonghai Lake (38°54′ N, 112°14′ E), which lies in northern Shanxi Province, China (Figure S1). Sampling was carried out using a custom-made PVC sampling corer (height: 20 cm; diam.: 4 cm) in January 2022. The sediment cores were collected from three different depths, namely, S1 (0–5 cm deep), S2 (5–10 cm deep) and S3 (10–20 cm deep). Water samples (namely, W) were collected from the overlying water at a height of 0–10 cm above the sampling point for the sediment core. These 24 samples were placed on ice and immediately transported to the laboratory within 24 h using a portable refrigerator. The samples were divided into two subsamples for analyzing physicochemical properties and DNA extraction (stored at −80 °C).

The following physicochemical factors were measured according to previously described methods [18,20,25]. Environmental properties such as water pH, ammonium (NH_4_^+^-N) and nitrate (NO_3_^−^-N) content were monitored at the sampling site using a portable multiparameter water monitoring probe (Aquaread AP-5000, Broadstairs, UK). Total organic carbon (TOC), total carbon (TC) and inorganic carbon (IC) were analyzed using a TOC analyzer (Shimadzu, TOC-VCPH, Shimane, Japan). Sediment pH was determined in 1 M KCl sediment suspension [sediment: water ratio of 1:2.5 (*w/v*)]. NH_4_^+^-N and NO_3_^−^-N were determined by automated discrete analysis (CleverChem 380, Hamburg, Germany). TC was determined by elemental analysis (Elementar Vario MACRO, Frankfurt, Germany). Organic carbon (SOC) was measured using the K_2_Cr_2_O_7_ oxidation method.

### 2.2. DNA Extraction and Sequencing

Microorganisms in water samples were collected by filtration through a 0.2 μm pore size membrane filter (Millipore, Jinteng, Tianjin, China). The biomass-containing filters and sediment samples were used for DNA extraction using FastDNA SPIN kits (Omega Biotek, Norcross, GA, USA). The specific conditions of PCR amplification and purification can be found in our previous work [25]. The primers 338F (5′-ACTCCTACGGGAGGCAGCAG-3′) and 806R (5′-GGACTACHVGGGTWTCTAAT-3′) were used to amplify the V3–V4 region of the 16S rRNA bacterial gene. The variable region of the ITS genes was amplified using the universal forward primer ITS1F (5′-CTTGGTCATTTAGAGGAAGTAA-3′) and universal reverse primer ITS1R (5′-GCTGCGTTCTTCATCGATGC-3′). The V4 region of the 18S rRNA gene was amplified using the primers TAReuk454FWD1F (5′-CCAGCASCYGCGGTAATTCC-3′) and TAReukREV3R (5′-ACTTTCGTTCTTGATYRA-3′). After the purification and quantification of the PCR products, genomic DNA libraries were constructed on an Illumina MiSeq platform (Majorbio Bio-Pharm Technology Co., Ltd., Shanghai, China). The sequencing reads of fungi, metazoans, archaea and unclassified sequences were removed from the 18S rRNA sequences. Detailed steps of the data processing can be found in the previous study [18]. The number of OTUs (Operational Taxonomic Units) was analyzed for each sample using a 97% sequence similarity cutoff value.

### 2.3. Statistical Analysis

Microbial diversity (Sobs and Shannon index) was calculated using the R package ‘vegan’ (v. 4.1.3). Additionally, the variation in community composition was evaluated by NMDS (Non-metric multidimensional scaling) based on the Bray–Curtis distance matrix. The significant differences between depths were determined by ANOSIM. The Sobs (OTUs), Shannon diversity and composition (two beta-NMDS axes) of fungi, protozoa and algae represented biotic factors. The effects of abiotic factors and biotic diversity on the diversity of bacterial community and the abundance of important taxa were estimated by correlation and best multiple regression model analyses in the R packages ‘psych’ (v. 2.3.9) and ‘reshape2’ (v. 1.4.4), which factors were retained for forward select analysis. Mantel and partial Mantel tests (‘ecodist’ package) were used to assess the effects of all variations in the community structure. The random forest regression model was calculated in the ‘randomForest’ R package (4.7-1.1). Microbial OTUs were identified by Random Forests based on their relative abundances against sample depth. This model was further refined by the function ‘rfcv’ with tenfold cross-validation based on the percentage increase in the mean-squared error. In this refined model, OTUs were arranged in descending hierarchy of their importance to the accuracy of this model. This importance was determined based on the percentage increase in the mean-squared error of sample depth prediction when the relative abundance values of each taxon were randomly permuted. The assembly of bacterial communities was estimated by Null model analysis [8]. The beta nearest taxon index (*β*NTI) was calculated in the R package ‘picante’ (v. 1.8.2) with the function ses.mntd based on the phylogenetic tree with 999 randomization across all samples. Bray–Curtis dissimilarity (RC_bray_) was calculated in the R packages ‘vegan’ (v. 2.6-4) and ‘parallel’ (v. 4.1.3). Based on this, the *β*NTI and RC_bray_ values were combined to categorize assembly processes (homogeneous selection, heterogeneous selection, dispersal limitation, homogenizing dispersal and undominated) [26]. To explore potential interactions between bacterial and eukaryote (fungi, protozoa and algae) taxa, we constructed interdomain ecological networks (bipartite matrix co-occurrence networks). Spearman’s correlation coefficients were calculated in the R packages ‘psych’ (v. 2.3.9) and ‘reshape2’ (v. 1.4.4) (|r| > 0.6, *p* < 0.05), and then constructed using and visualized in Gephi (version 0.9.2). In addition, robustness indices (package patchwork, v.1.1.2) were employed to reflect the networks’ stability. The impact of biotic and abiotic factors on the assembly of bacterial communities was comprehensively analyzed through the partial least squares path modeling (PLS-PM) using the R package ‘plspm’. After removing the variables with loadings < 0.7 and performing the final PLS-PM structure equation with the remaining variables, the prediction performance of the model was evaluated using the goodness of fit index (Gof) and R^2^. The confidence interval of all statistical analyses was 95% (*p* < 0.05).

## 3. Results and Discussion

### 3.1. The Composition of the Microbial Communities Across the Sediment–Water Interface

**Figure 1:**
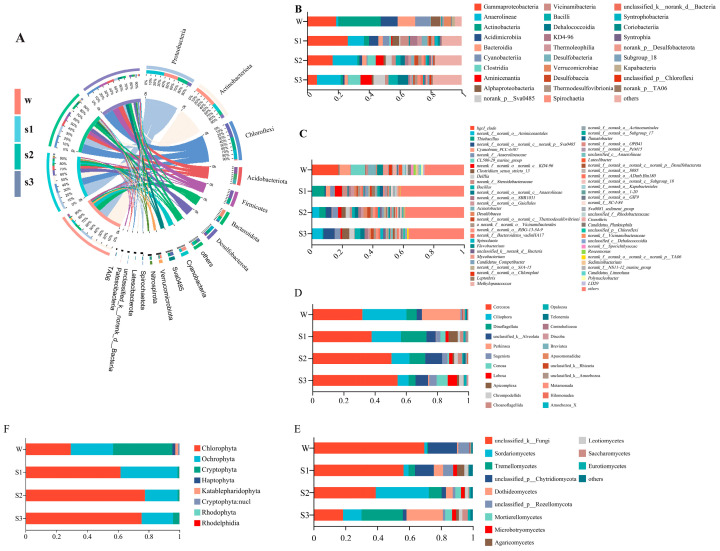
Composition of bacterial communities at phylum (**A**), class (**B**) and genera (**C**) levels in overlying water (W), surface sediment (S1), middle sediment (S2) and bottom sediment (S3) in GH lake, as well as the composition of protozoan (**D**), fungal (**E**) and algal (**F**) communities at phylum levels.

The composition of the microbial community varied between the sediments and the overlying water (Figure 1), which means that there were differences in the microbial community in this cross habitat [27,28]. The composition in the overlying water and sediment at different depths were clearly separated based on the first two axes of NMDS (Figure S2A, *p* < 0.001). At the class level, the relative abundances of Actinobacteria, Bacteroidia, Acidimicrobiia, Cyanobacteriia, Alphaproteobacteria and Verrucomicrobiae were higher in overlying water (27.51%, 11.35%, 10.91%, 9.79%, 5.96% and 4.22%, respectively) than in sediments (2.05%, 2.67%, 2.91%, 1.67%, 2.32% and 0.46%, respectively). These classes are known to participate in biogeochemical cycles in the water environment [29]. For instance, Actinobacteria is a main decomposer of organic carbon [30], and Bacteroidetes are known for their ability to degrade complex polysaccharides, making them important players in the carbon cycle [31]. The genera in overlying water were dominated by *hgcI_clade* (17.81%), *CL500-29_marine_group* (9.49%), *Cyanobium_PCC-6307* (7.33%), *Acinetobacter* (5.01%) and *Flavobacterium* (3.7%). *HgcI_clade* belongs to Actinobacteria, a typical freshwater cluster, performs aerobic-heterotrophic metabolism and is widespread in oligotrophic freshwater [32].

The taxonomic composition of bacterial communities across the sediment layer was significantly different. Proteobacteria was the dominant phylum in surface sediment (30.79%), which plays an important role in the degradation and metabolism of carbon and nitrogen in lake sediments [33]. Gammaproteobacteria was the dominant class in surface sediment (26.38%). The abundance of *Delftia* in deep sediments was greater than that in overlying water, which can reduce nitrate to nitrite [34]. Chloroflexi was the dominant phylum in bottom sediment, participating in the carbon, nitrogen and sulfur biogeochemical cycling, as well as performing both aerobic and anaerobic respiration in terms of energy metabolism [35]. Additionally, the abundance of classes of Anaerolineae (10.43%, 15.94% and 15.36%), Clostridia (1.23%, 5.30% and 7.89%), norank_p_Sva0485 (0.82%, 3.86% and 7.49%), Aminicenantia (0.75%, 5.07% and 7.26%), Dehalococcoidia (0.34%, 2.78% and 6.69%) and Bacilli (0.42%, 3.09% and 6.03%) increased with sediment depth. Aminicenantia appear adapted to scavenge organic carbon, as each has genetic potential to catabolize amino acids and fatty acids [36]. These results indicated that bacterial taxa have their own unique habitat preferences at the phylum, class and genus levels, each specialized in carrying out the metabolism of carbon, nitrogen and sulfur within their respective habitat.

The composition of the protozoa community in the overlying water and sediment at different depths was clearly separated based on the first two axes of NMDS (Figure S2C, *p* < 0.001). Cercozoa was the richest and most abundant taxon in sediments and overlying water and more abundant in deep than shallow sediments on average (Figure 1D). Ciliophora was abundant in overlying water, while Dinoflagellata was abundant in surface sediment. Trophic status was assigned for each taxon within the protozoa communities on a genus level (> 10% of total abundance), which included consumers (50.24%), parasites (20.29%), photoautotrophs (4.35%) and unassigned (25.11%) (Table S1). Meanwhile, consumers included the bacterivore group (41.19%), eukaryvore group (34.76%), omnivore group (19.23%) and saprotroph group (1.42%). Among consumers, the genus of *g_Prototaspa lineage_X* (Eukaryvore group, 21.8%) had a higher abundance in overlying water, and *g_Leptophryidae_X* (Eukaryvore group, 3.33%), *g_Allantion* (Bacterivore group, 8.33%) and *g_unclassified_f_Sandonidae* (Bacterivore group, 10.7%) were the dominant genera in the surface, middle and bottom sediment, respectively. These consumers are involved in regulating the structure of bacterial and fungal communities in natural ecosystems [37]. For the fungal communities, unclassified_k_Fungi, unclassified_p_Chytridiomycota and unclassified_p_Rozellomycota dominated the phylum in the overlying water. Saccharomycetes was the dominant phylum in the surface sediment. Sordariomycetes dominated in the middle sediment, while Tremellomycetes and Dothideomycetes dominated in the bottom sediment (Figure 1E). For algal communities, Chlorophyta and Ochrophyta were the dominant phyla across all habitat layers; Cryptophyta dominated in the overlying water (Figure 1F).

### 3.2. Drivers of the Diversity of the Bacterial Community Across the Sediment–Water Interface

**Figure 2:**
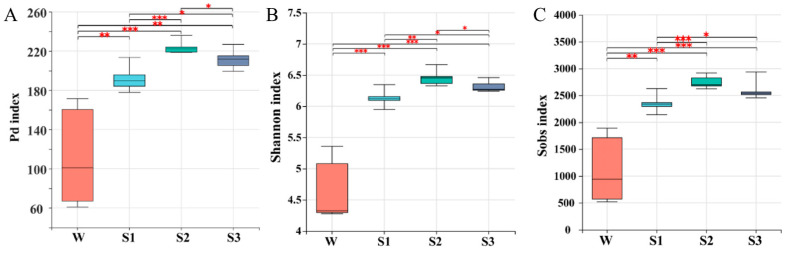
The bacterial phylogenetic diversity (measured by the Faith index) (**A**), *α*-diversity (represented by the Shannon index (**B**) and Sobs index (**C**)) and its difference in bacterial communities at the overlying water (W), surface sediment (S1), middle sediment (S2) and bottom sediment (S3) in GH lake. “*” represents the degree of significance [*p* < 0.001 (***), *p* < 0.01 (**), *p* < 0.05 (*)].

The Shannon and Sobs index (6.299 and 2563.111) and Phylogenetic diversity (208.965) of bacterial community were slightly higher in sediment than that (4.649, 1118.167 and 111.683, respectively) in overlying water (*p* < 0.01) (Figure 2). The bacterial diversity in the middle sediment was significantly higher than that in the bottom and surface sediments (*p* < 0.01). The diversity index of fungal and protozoan communities was highest in the surface sediment (0–5 cm), while that of the alga community was higher in overlying water than that in sediments (Figure S3). Most microorganisms in sediments were recruited from surroundings [38], while in this study, bacteria had higher diversity than alga, fungus and protozoan in the deeper sediments. This means that bacteria are better able to tolerate oxygen-deficient and dark environments in sediment compared to eukaryotic organisms.

**Figure 3:**
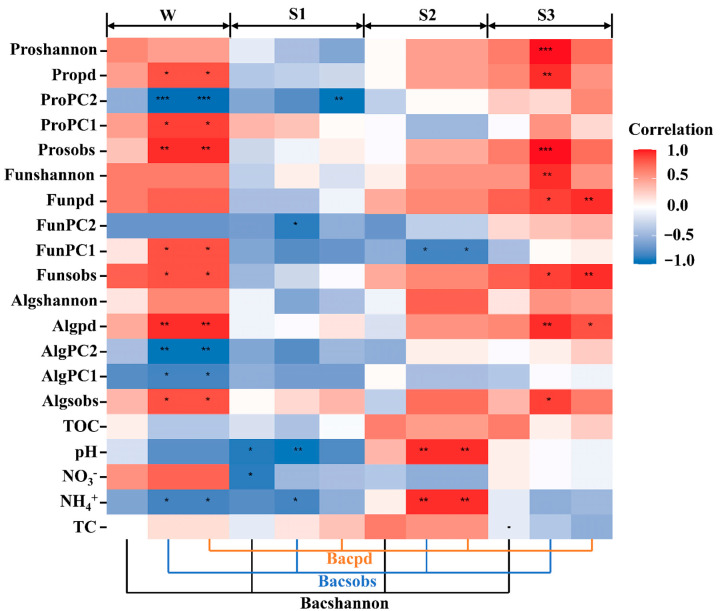
Contributions of eukaryote community composition variables and physicochemical parameters to the diversity of bacterial communities based on correlation analysis in overlying water (W), surface sediment (S1), middle sediment (S2) and bottom sediment (S3). Colors represent Spearman correlations. Community composition variables in the model were a combination of microbial *α*-diversity, phylogenetic diversity (measured by Faith’s index), the first axis score of NMDS (NMDS1) and the second axis score of NMDS (NMDS2). “*” represents the degree of significance [*p* < 0.001 (***), *p* < 0.01 (**), *p* < 0.05 (*)].

Differences in microbial diversity have often been found between the water and sediment in various aquatic ecosystems, including river, lake and marine environments [14], where environmental factors play important roles. The pH and the concentration of NH_4_^+^ and NO_3_^−^ in sediment increased progressively with depth (Figure S4). Pearson’s correlation analysis showed that the relationship between the bacterial community diversity and NH_4_^+^ concentration was significantly negatively correlated in overlying water and surface sediments while significantly positively correlated in middle sediments. And pH showed a similar trend in surface and middle sediments (*p* < 0.1) (Figure 3). These results support our first hypothesis that environmental heterogeneity is the main driver of the diversity of the bacterial community in cross-habitat ecosystems. In the middle sediment, the pH was about 8.5, which was suitable for the growth of microorganisms. In the middle sediment, the organic matter was lower than that in the surface sediments, leading to an overall slowdown in the aerobic heterotrophic respiration and the ammonification (Figure S4A). It created a better living environment for the growth of other aerobic microorganisms, thereby increasing the diversity of microorganisms in the middle sediment. In the bottom sediment, there was more organic matter than in the surface sediments and middle sediment (Figure S4A). The varying trends of TOC and nitrogenous compounds in the sediment at different depths indicated that the organic matter in the bottom sediment might be mainly composed of recalcitrant organic matter (Figure S4A,D,E,F). Many microorganisms have difficulty utilizing these recalcitrant organic compounds for energy metabolism, which reduces the diversity of microbial communities in the bottom sediment. Furthermore, the high pH and ammonia nitrogen resulting from intense denitrification and ammonification processes generated a significant amount of free ammonia, which also led to a reduction in microbial diversity (Figure S4E,G) [39].

On the other hand, there was a significant correlation between the diversity of bacterial communities and the composition variables of protist communities in overlying water and a significant positive correlation between them in bottom sediment (*p* < 0.05) (Figure 3). The interactions between algae and bacteria, such as mutualism or confrontation, were formed through the exchange of primary and secondary metabolites provided by one of the partners. Algae can secrete some special substances (organic acids, sugars and O_2_) to create habitats for bacteria, while bacteria can support algae growth through nutrient cycling. In addition, the growth and decomposition of algae may lead to an increase in pH, which in turn promotes bacterial growth [40]. In terms of predation regulation, the ratio of bacterivore groups to bacterial community diversity was the highest in the bottom sediment (Table S1). Among the 16 genera of bacterivore groups, 8 genera had the highest abundance in the bottom sediment, which is much higher than in other depths. The ratio of omnivore groups and bacterial communities in the overlying water was the highest. These results indicated that the protozoan community had a significant impact on the diversity of bacterial communities in the overlying water and bottom sediment. This supports the second hypothesis that protozoa have a greater impact on the diversity of the bacterial community across the sediment–water interface.

### 3.3. The Assembly of the Bacterial Community Across the Sediment–Water Interface

**Figure 4:**
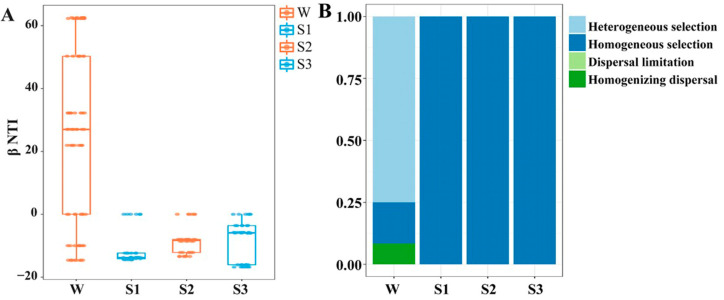
Assembly processes of bacterial communities. (**A**) *β*NTI value based on a null model to determine the stochastic and deterministic processes of bacterial community assembly; (**B**) The relative contributions of five processes (heterogeneous selection; homogeneous selection; dispersal limitation; homogenizing dispersal; undominated process), based on *β*NTI and RC_Bray_ in assembly processes of bacterial communities.

The abundance-weighted beta nearest taxon index (*β*NTI) analyses showed that selection was one of the essential processes in bacterial community assembly (Figure 4A). Null model analysis further revealed that heterogeneous selections were the primary driver of community assembly in overlying water, followed by homogeneous selection and homogenizing dispersal, whereas homogeneous selection was more important in sediments (Figure 4B). Consistent with studies in other subsurface systems, deterministic processes dominated the microbial community assembly at the full-sediment system scale [13]. Overall, the assembly processes of bacterial communities in overlying water were more complex than in sediments. There was also a strong relationship between *β*NTI and depth, implying that the determinacy of bacterial community assembly was influenced by the habitat difference (*p* < 0.001) (Figure S5). Zhou et al. showed that lowly permeable sediments restricted vertical water exchange and imposed dispersal limitation and selection [41]. Limited nutrient conditions in sediments are likely to exert a more stringent limitation on microbial survival and fitness, imposing strong selective pressures. This might explain why homogeneous selection played more important roles in deeper sediment. Comparatively, overlying water provided channels to promote the active and passive dispersal of microorganisms, probably accounting for the dispersal limitation there [42].

### 3.4. Effects of Biological Factors on Bacterial Community Assembly Across the Sediment–Water Interface

**Figure 5:**
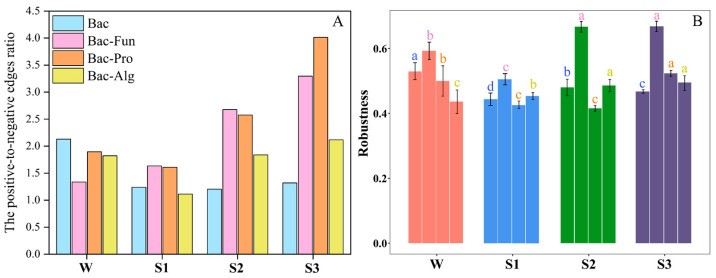
(**A**) The positive-to-negative edges ratio of bacterial communities, bacterial communities with fungus (Bac-Fun) and protozoan (Bac-Pro) and alga (Bac-Alg) communities in W, S1, S2 and S3. (**B**) Robustness of bacterial communities, bacterial communities with fungus (Bac-Fun) and protozoan (Bac-Pro) and alga (Bac-Alg) communities in W, S1, S2 and S3. Different letters above the error bar indicate statistical difference among the robustness of four microbial communities (*p* < 0.05).

Interactions among microorganisms are complicated and important factors for community assembly [43]. To analyze microbial interactions across the sediment–water interface, we selected the bacterial, fungal, alga and protozoan communities to construct their co-occurrence bipartite networks. The positive-to-negative edges ratios of the bacterial community with fungal, protozoan and alga communities were 2.23 (P/N-BF), 2.50 (P/N-BP) and 1.72 (P/N-BA), respectively, suggesting significant differences in the biotic interactions between bacteria and each microeukaryote (Figure 5A). The robustness of the bacterial community network was higher in overlying water than in sediments, while the robustness of the bipartite network was significantly higher in the bottom sediment than others (Figure 5B). Especially, the robustness of the bacterial–fungal community bipartite network was significantly higher than others (*p* < 0.01), implying that fungus may play a major role in maintaining the stability of the microbial network across the sediment–water interface [44].

**Figure 6:**
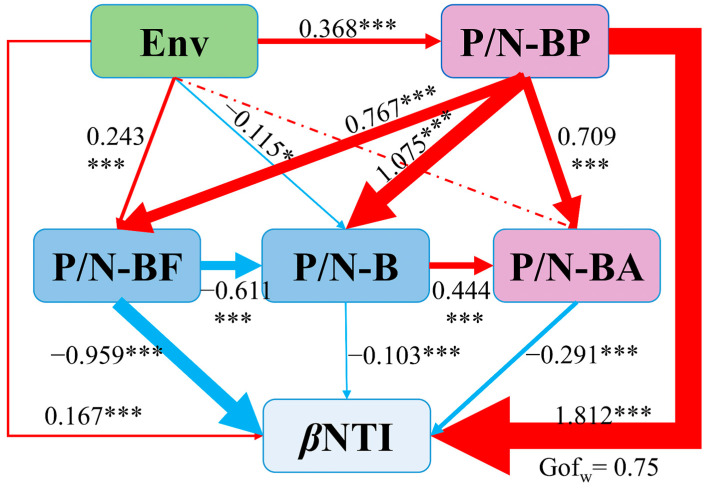
Effects of different variables on the assembly of bacterial communities (*β*NTI value) based on partial least squares path modeling. The red arrows represent positive pathways and the blue arrows indicate negative pathways. The positive-to-negative edges ratio (P/N) denoted biological factors; P/N-BP, P/N-BF and P/N-BA represent the positive-to-negative edges ratio of the bacterial community with protozoan, fungal and alga communities. Env represent the environmental heterogeneity (based on Euclidean Distance). The path coefficients are shown on the arrow. GOF, goodness of fit. “*” represents the degree of significance [*p* < 0.001 (***), *p* < 0.05 (*)].

The partial least squares path model (PLS-PM) indicated a significant correlation between the environmental heterogeneity, biotic variables and *β*NTI of the bacterial community across the sediment–water interface (*p* < 0.001) (Figure 6). In line with our initial expectations, the strongest relationship is the biotic interaction of protozoan and bacterial communities (P/N-BP) with *β*NTI, while the environmental heterogeneity is less significant, suggesting the important effect of protozoan on the assembly of the bacterial community. This could be explained as the top–down predation of protozoa, where predation behavior can release nutrients from the prey, thus promoting the ecosystem’s element cycling and energy flow [45]. Protozoan predation represents a major selection pressure that bacteria face in both natural and anthropogenic environments. Bacterivorous protozoa, such as ciliates, amoebae and flagellates, tightly regulate bacterial populations [46], which might affect the assembly of bacterial communities. The predation prevented competitive exclusion, and the increase in consumers’ abundance increased the prey’s evenness and diversity, thereby affecting its structural stability, especially in aquatic environments [47]. It can explain the impact of protozoa–bacterial trophic interaction on bacterial community assembly. We found that fungal–bacterial interaction also significantly affected the assembly of bacterial communities (Figure 6). Previous studies showed that negative bacterial–fungal associations tended to be lower when communities were driven primarily by neutral processes [48]. This means that positive bacterial–fungal associations were more widespread under stronger stochastic processes. These findings demonstrated that the nontrophic interactions between eukaryote and bacterial communities could act as a kind of deterministic selection force for bacterial community assembly.

### 3.5. Effects of Important Taxa on the Assembly Process of the Bacterial Community

The important taxa of bacterial and microeukaryotic communities in the sediment–water interface were identified by random forest regression analysis (Figure S7). Two hundred and eighty bacterial OTUs were ranked by importance to the accuracy of the model. The most abundant taxa were OTU4495 (p_Firmicutes), OTU6982 (p_Proteobacteria) and OTU8149 (p_Acidobacteriota).

We found large changes in nodes and links among co-occurrence networks of different layers, suggesting differences in microbial interactions among overlying water and sediments, especially the significant difference in core species, which were defined as those that had both a high connectivity and degree in the network [44]. Interestingly, there was an overlap between core species and important taxa at the sediment–water interface, suggesting that these important taxa played a significant role in regulating the relationships between biotic interactions (Figure S8). For example, OTU4695, an important taxon in overlying water, belonging to the genus of Cyanobium_PCC-6307 with high abundance, was screened as an important node in the co-occurrence network (Betweenness centrality was 1015.730) (Figure S8). OTU781, belonging to the Thiobacillus genus with high abundance, has been screened as an important OTU in both water and sediment and is also an important node in the co-occurrence network (Betweenness centrality was 698.115). The multiple regression model analysis showed that the physicochemical parameters and composition variables of fungal and protozoan communities had an impact on the abundance of important taxa in the overlying water, surface and middle sediments, while composition variables of algae, protozoan and fungal communities had an impact on that in the bottom sediment (Figure S9).

**Figure 7:**
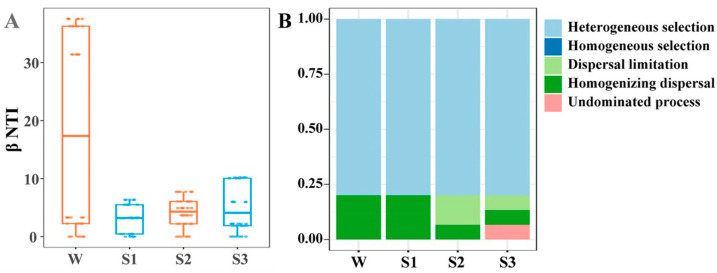
Assembly processes of bacterial communities after removing important OTUs. (**A**) *β*NTI value based on the null model to determine the stochastic and deterministic processes of bacterial community assembly after removing important OTUs; (**B**) The relative contributions of five processes (heterogeneous selection; homogeneous selection; dispersal limitation; homogenizing dispersal; undominated process) based on *β*NTI and RC_Bray_ in assembly processes of bacterial communities after removing important OTUs.

To verify whether important taxa have an impact on the assembly of bacterial communities, we removed important taxa from the bacterial community to conduct the null model analysis. After removing important taxa, we observed that the values of *β*NTI were all above zero, leading to significant changes in the assembly processes of bacterial communities across the sediment–water interface (Figure 7). This contrasts significantly with the non-removal ones where the values of *β*NTI in sediments are far less than zero (Figure 4). The relative importance of homogenous dispersal in the assembly process of the bacterial community in overlaying water has increased. And heterogeneous selections have replaced homogeneous selection, becoming the dominant process of that in sediments. These results indicate that the impact of biotic interactions on bacterial community assembly may be related to the important taxa.

Biotic interactions drive population dynamics by influencing the growth and mortality, competition and predation of taxonomic units. It could be a powerful driving force for the assembly of microbial communities, comparable to or surpassing the influence of environmental factors [37,49]. The important taxa constrain community assembly through biotic interactions with other taxa. Researchers have shown that keystone taxa are involved in regulating the composition of microbial communities and can affect community assembly through strong biological interactions [50]. Thus, as theoretically deduced in this study, removing important taxa may lead to rapid shifts of dominant processes in community assembly.

## 4. Conclusions

There exist significant differences among the structures of microbial communities in cross habitats from overlying water to sediments in the subalpine GH lake. The physicochemical properties, as the main driver across the sediment–water interface, shape the diversity and important taxa of the microbial community. The distinctness of assembly processes of bacterial communities among the sediment–water interface can be explained by combinations of environmental heterogeneity and biotic interactions, where the biotic interaction between protozoa and bacterial communities had the greatest impact. Important taxa influence the assembly of bacterial communities by regulating biotic interactions in a co-occurrence network. These results may provide references, supplements and indications for the assembly processes of subalpine lakes worldwide. The data in this study are only sourced from a subalpine lake, and the sample size is limited, which inevitably leads to certain limitations in the conclusions. We expect to have more data at a larger scale to validate this conclusion.

## Data Availability

The original contributions presented in the study are included in the article/Supplementary Materials, further inquiries can be directed to the corresponding author.

## Supplementary Materials

The following supporting information can be downloaded at: https://www.mdpi.com/article/10.3390/microorganisms12122418/s1, Figure S1: Schematic diagram of the Gonghai subalpine lake and locations of sampling sites; Figure S2: NMDS (Non-metric multidimensional scaling) of bacterial (A), fungus (B), protozoan (C) and alga (D) communities at the overlying water (W), surface sediment (S1), middle sediment (S2) and bottom sediment (S3) in GH lake; Figure S3: The phylogenetic diversity (measured by the Faith index), microbial *α*-diversity (represented by the Shannon index and Sobs index) and its difference in fungus (A), protozoan (B) and alga (C) communities at the overlying water (W), surface sediment (S1), middle sediment (S2) and bottom sediment (S3) in GH lake. “*” represents the degree of significance [*p* < 0.001(***), *p* < 0.01(**), *p* < 0.05(*)]; Figure S4: Physicochemical parameters at the surface sediment (S1), middle sediment (S2) and bottom sediment (S3) in GH lake. (A) TOC: Total organic carbon; (B) TC: Total carbon; (C) TS: Total sulphur; (D) TN: Total nitrogen; (E) NH_4_^+^: Ammonium nitrogen (F) NO_3_^−^: nitrate nitrogen; (G) pH. “*” represents the degree of significance [*p* < 0.001(***), *p* < 0.01(**), *p* < 0.05(*)]; Figure S5: The results of the linear least-squares regression analysis for depth difference based on *β*NTI in GH lake; Figure S6: (A) Physicochemical parameters of W, S1, S2 and S3. (B) The differences in environmental heterogeneity between different sites are significantly distinguished from the differences within the sample site (based on Euclidean Distance). Different letters above the error bar indicate statistical difference among the robustness of four microbial communities (*p* < 0.05); Figure S7: Rank importance of OTUs determined by applying the random forest regression to the microbial community of the water sediment interface. The importance of OTUs is determined by the percentage increase in the mean squared error of microbiota prediction when the relative abundance of each OTU was randomly permuted (mean importance ± s.d., n = 100 replicates). OTUs are colored based on the phylum level. A, Two hundred and eighty bacterial OTUs ranked by importance to the accuracy of the model. The tenfold cross-validation error is also displayed in order of variable importance. The lowest error value represents the 280 OTUs used in the model. Heat map of mean relative abundance of the 280 selected OTUs in the water sediment interface. B, Fifty-eight OTUs of fungus ranked by importance to the accuracy of the model. C, Sixteen OTUs of protozoan ranked by importance to the accuracy of the model. D, Eight OTUs of alga ranked by importance to the accuracy of the model; Figure S8: Bipartite co-occurrence network of bacteria and eukaryote (fungal, protozoa and alga) in overlying water (A), surface sediment (B), middle sediment (C) and bottom sediment (D). The green lines depict negative correlation and red lines depict positive correlation, with a correlation > 0.6 and *p* < 0.05. The size of the node is proportional to its degree. Different taxa are shown in different colors in the bipartite co-occurrence network; Figure S9: Contributions of eukaryote community diversity (Shannon, sobs and pd index) and physicochemical parameters to the bacterial diversity and important OTUs based on the best multiple regression model in (A) overlying water (W), (B) surface sediment (S1), (C) middle sediment (S2) and (D) bottom sediment (S3). The bar chart represents the total contribution of biotic (Pro, Fun and Alg represent diversity of protozoa, fungal and alga communities, respectively) and abiotic indicators to explain bacterial and important OTU variation (proportion of explained variability calculated via multiple regression modeling). Circle size represents the variable importance (that is, the proportion of explained variability calculated via multiple regression modeling and variance decomposition analysis). Colors represent Spearman correlations; Table S1: Trophic status of each taxon within the protozoa communities on the genus level (> 10% of total abundance) in overlying water (W), surface sediment (S1), middle sediment (S2) and bottom sediment (S3).
