# Supplementary material for: Biotic Interaction Underpins the Assembly Processes of the Bacterial Community Across the Sediment–Water Interface in a Subalpine Lake
Source: Microorganisms. 2024 Nov 25;12(12):2418. doi: 10.3390/microorganisms12122418 (PMC11677085; doi:10.3390/microorganisms12122418)
Supplement: Supplementary file 1 [file microorganisms-12-02418-s001.zip › microorganisms-3254287-supplementary.pdf]

Supplementary materials

Biotic Interaction Underpins the Assembly Processes of the Bacterial Community Across the Sediment–Water Interface in a Subalpine Lake

Xue Wang [1,2], Jinxian Liu [1,2,*], Jiali Ren [1,2] and Baofeng Chai [1,2]

[1] Institute of Loess Plateau, Shanxi University, Taiyuan 030006, China; 202113202005@email.sxu.edu.cn (X.W.); 202323202021@email.sxu.edu.cn (J.R.); bfchai@sxu.edu.cn (B.C.)

[2] Shanxi Key Laboratory of Ecological Restoration for Loess Plateau, Shanxi University, Taiyuan 030006, China

* Correspondence: liujinxian@sxu.edu.cn; Tel.: +86-7010700

Number of pages: 12
Number of figures: 9
Number of table: 1

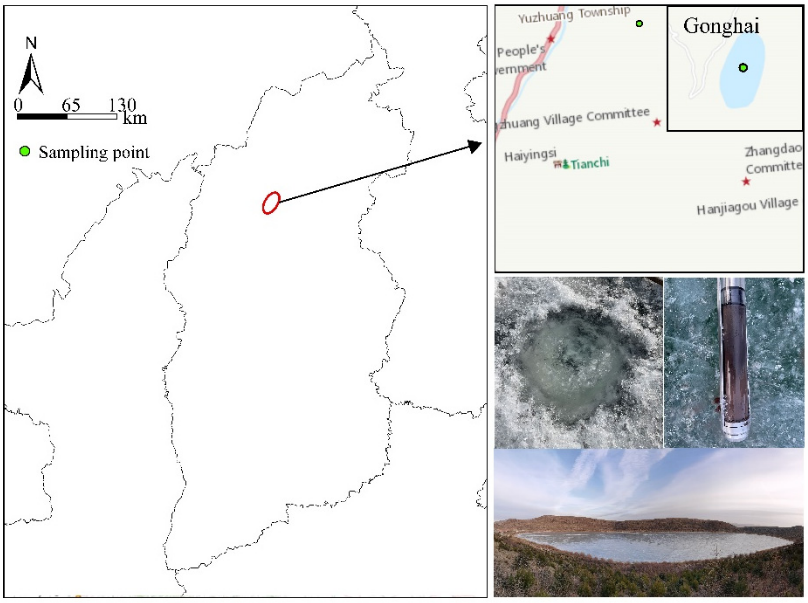

Figure S1. Schematic diagram of the Gonghai subalpine lake and locations of sampling sites.

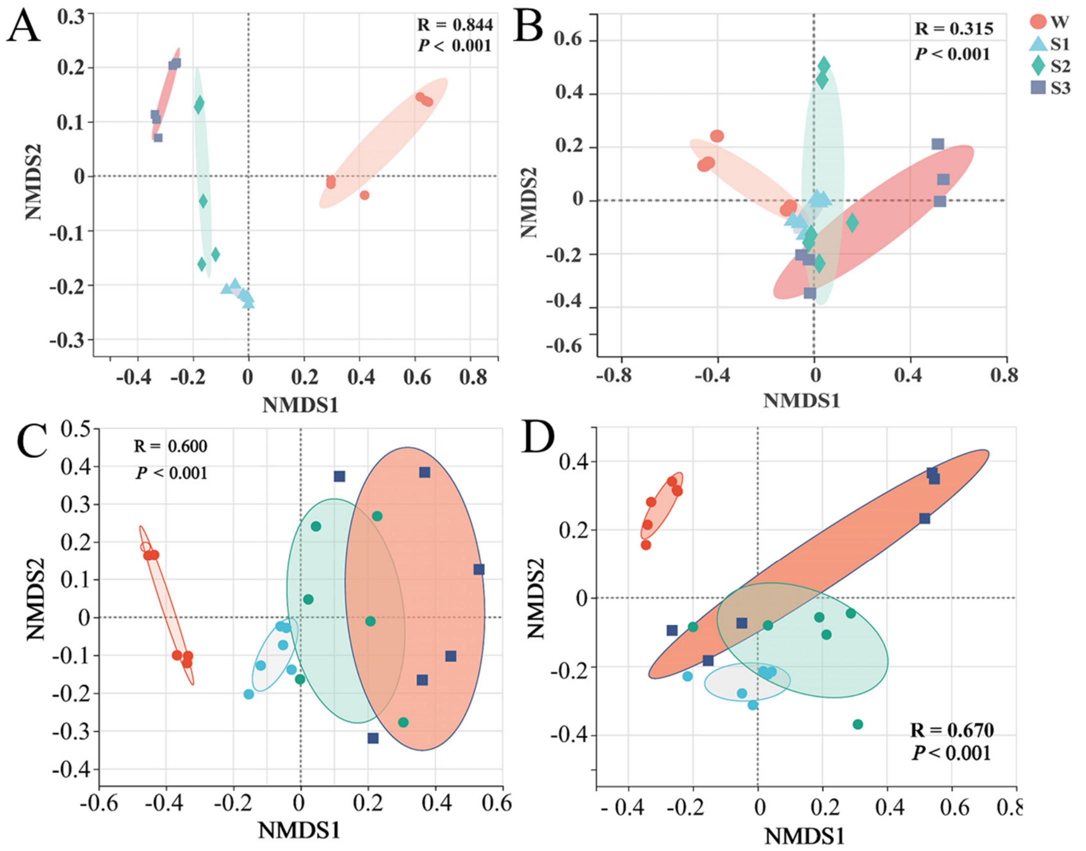

Figure S2. NMDS (Non-metric multidimensional scaling) of bacterial (A), fungus (B), protozoan (C) and alga (D) communities at the overlying water (W), surface sediment (S1), middle sediment (S2) and bottom sediment (S3) in GH lake.

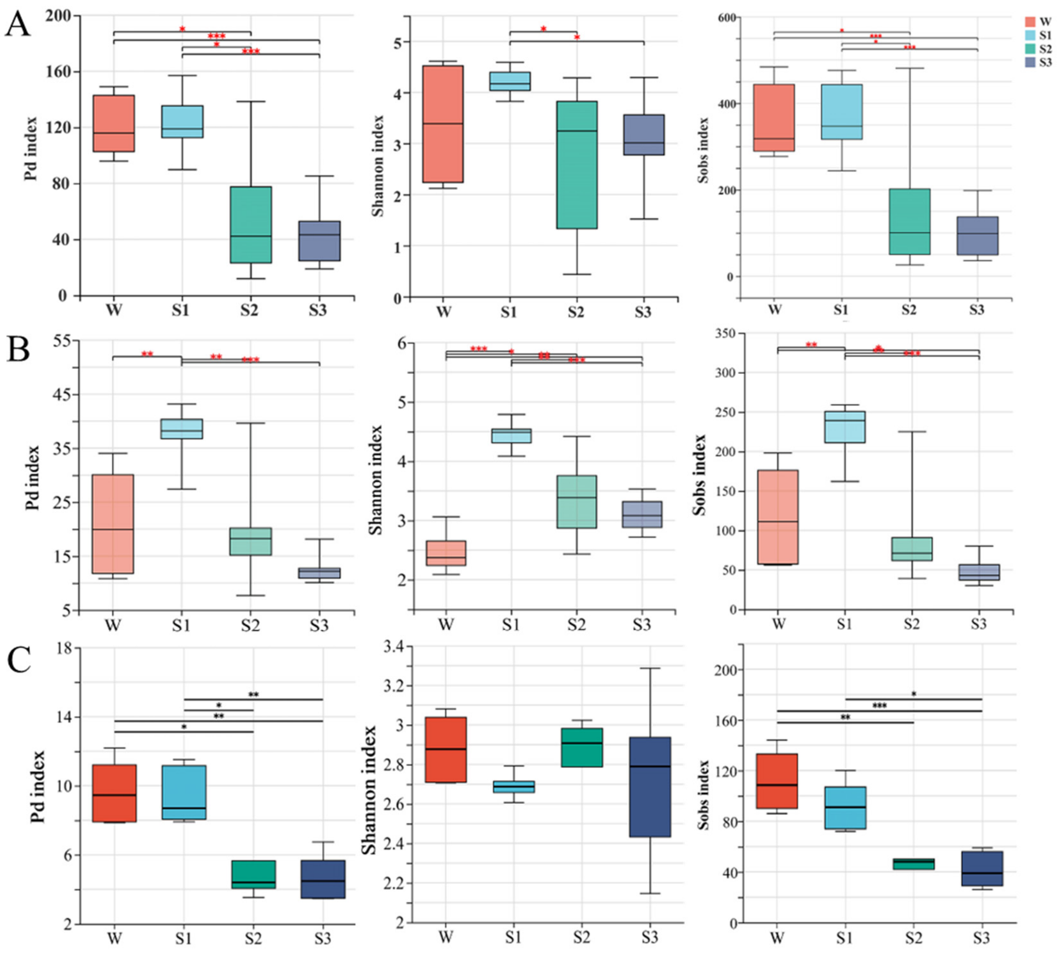

Figure S3. The phylogenetic diversity (measured by the Faith index), microbial α-diversity (represented by the Shannon index and Sobs index) and its difference in fungus (A), protozoan (B) and alga (C) communities at the overlying water (W), surface sediment (S1), middle sediment (S2) and bottom sediment (S3) in GH lake. "*" represents the degree of significance [$p < 0.001$(***), $p < 0.01$(**), $p < 0.05$(*)].

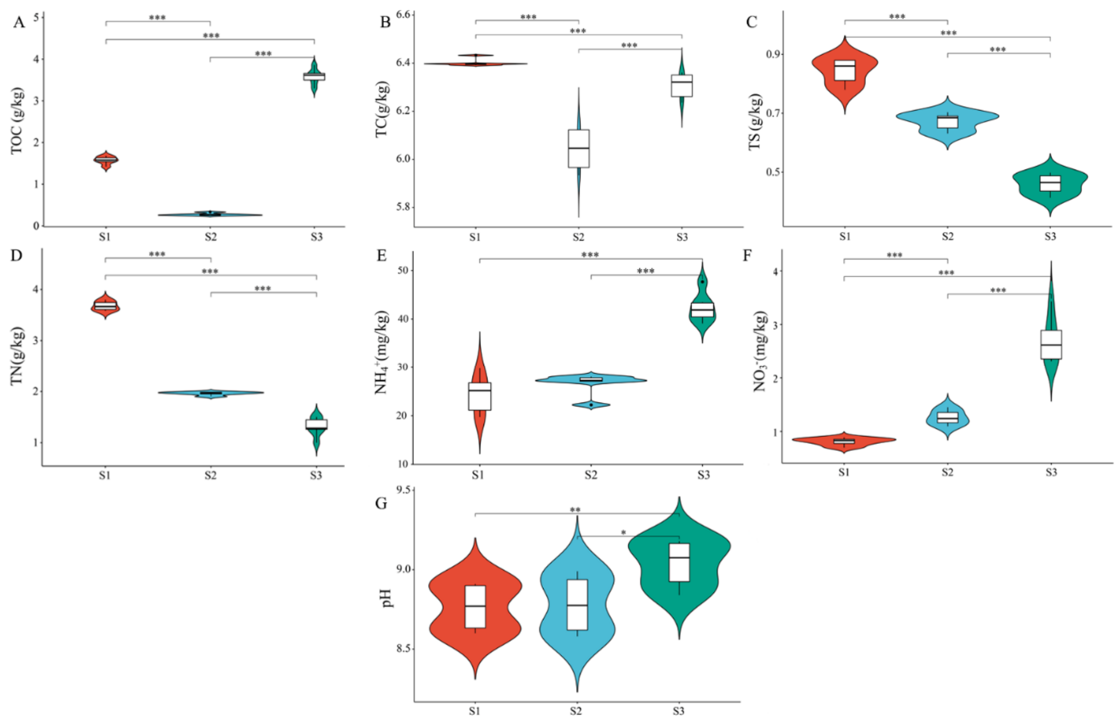

Figure S4. Physicochemical parameters at the surface sediment (S1), middle sediment (S2) and bottom sediment (S3) in GH lake. (A) TOC: Total organic carbon; (B) TC: Total carbon; (C) TS: Total sulphur; (D) TN: Total nitrogen; (E) NH_4^+: Ammonium nitrogen (F) NO_3^-: nitrate nitrogen; (G) pH. "*" represents the degree of significance [p < 0.001(***), p < 0.01(**), p < 0.05(*)].

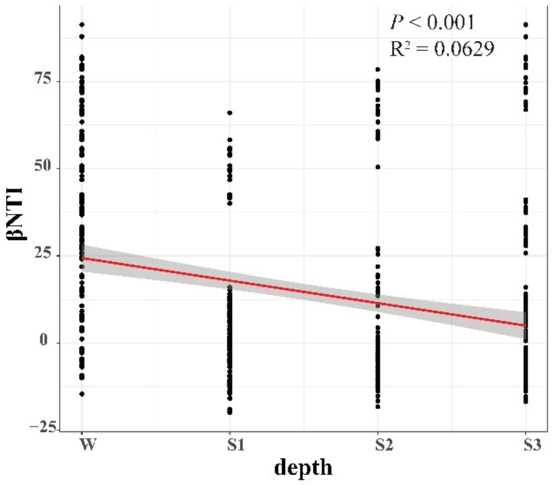

Figure S5. The results of the linear least-squares regression analysis for depth difference based on *β*NTI in GH lake.

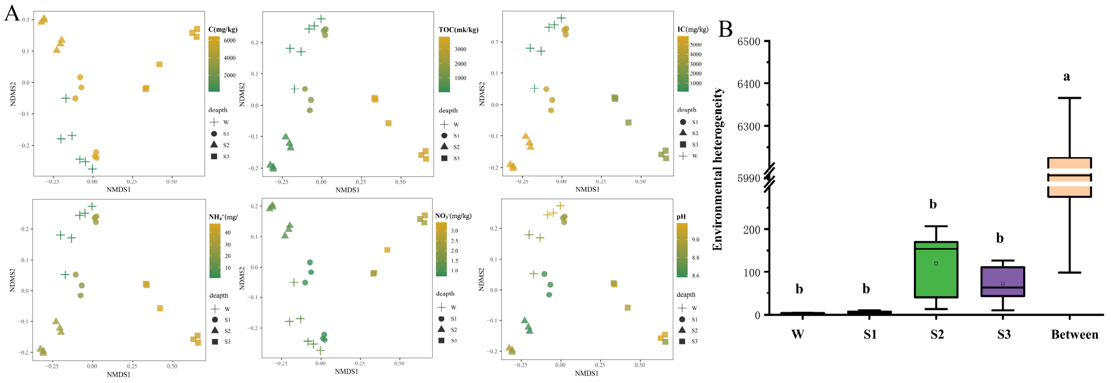

Figure S6. (A) Physicochemical parameters of W, S1, S2 and S3. (B) The differences in environmental heterogeneity between different sites are significantly distinguished from the differences within the sample site (based on Euclidean Distance). Different letters above the error bar indicate statistical difference among the robustness of four microbial communities ($p < 0.05$).

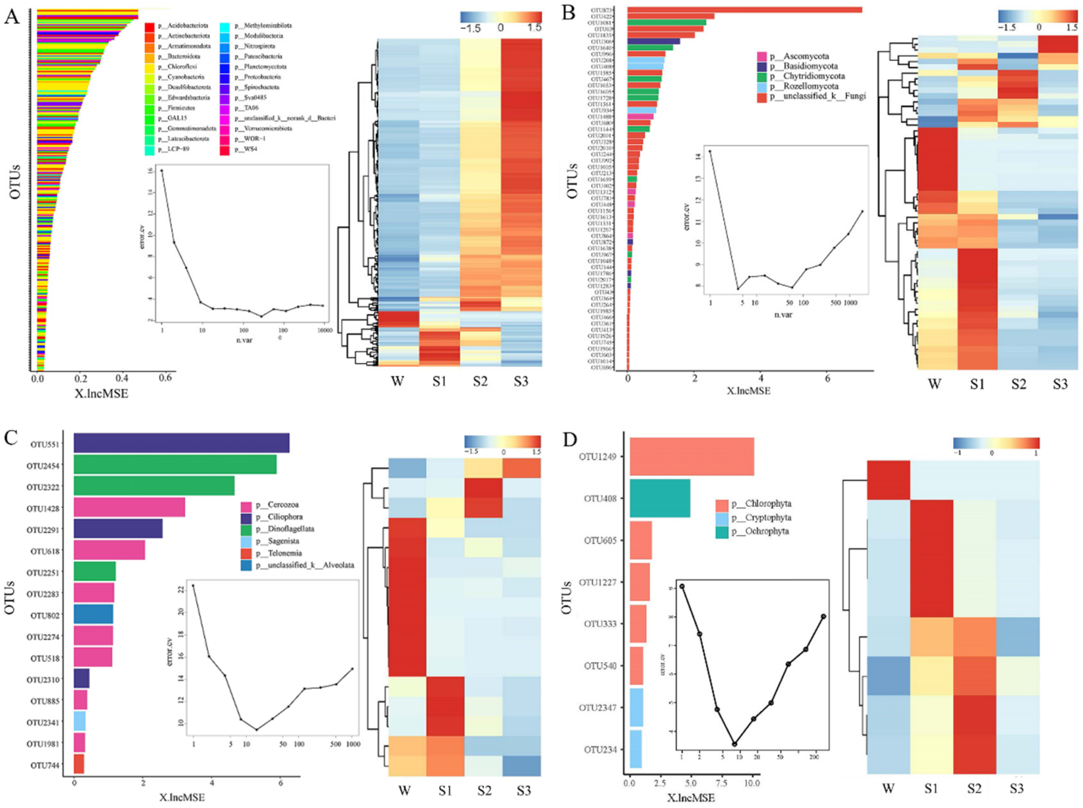

Figure S7. Rank importance of OTUs determined by applying the random forest regression to the microbial community of the water sediment interface. The importance of OTUs is determined by the percentage increase in the mean squared error of microbiota prediction when the relative abundance of each OTU was randomly permuted (mean importance ± s.d., n=100 replicates). OTUs are colored based on the phylum level. A, Two hundred and eighty bacterial OTUs ranked by importance to the accuracy of the model. The tenfold cross-validation error is also displayed in order of variable importance. The lowest error value represents the 280 OTUs used in the model. Heat map of mean relative abundance of the 280 selected OTUs in the water sediment interface. B, Fifty-eight OTUs of fungus ranked by importance to the accuracy of the model. C, Sixteen OTUs of protozoan ranked by importance to the accuracy of the model. D, Eight OTUs of alga ranked by importance to the accuracy of the model.

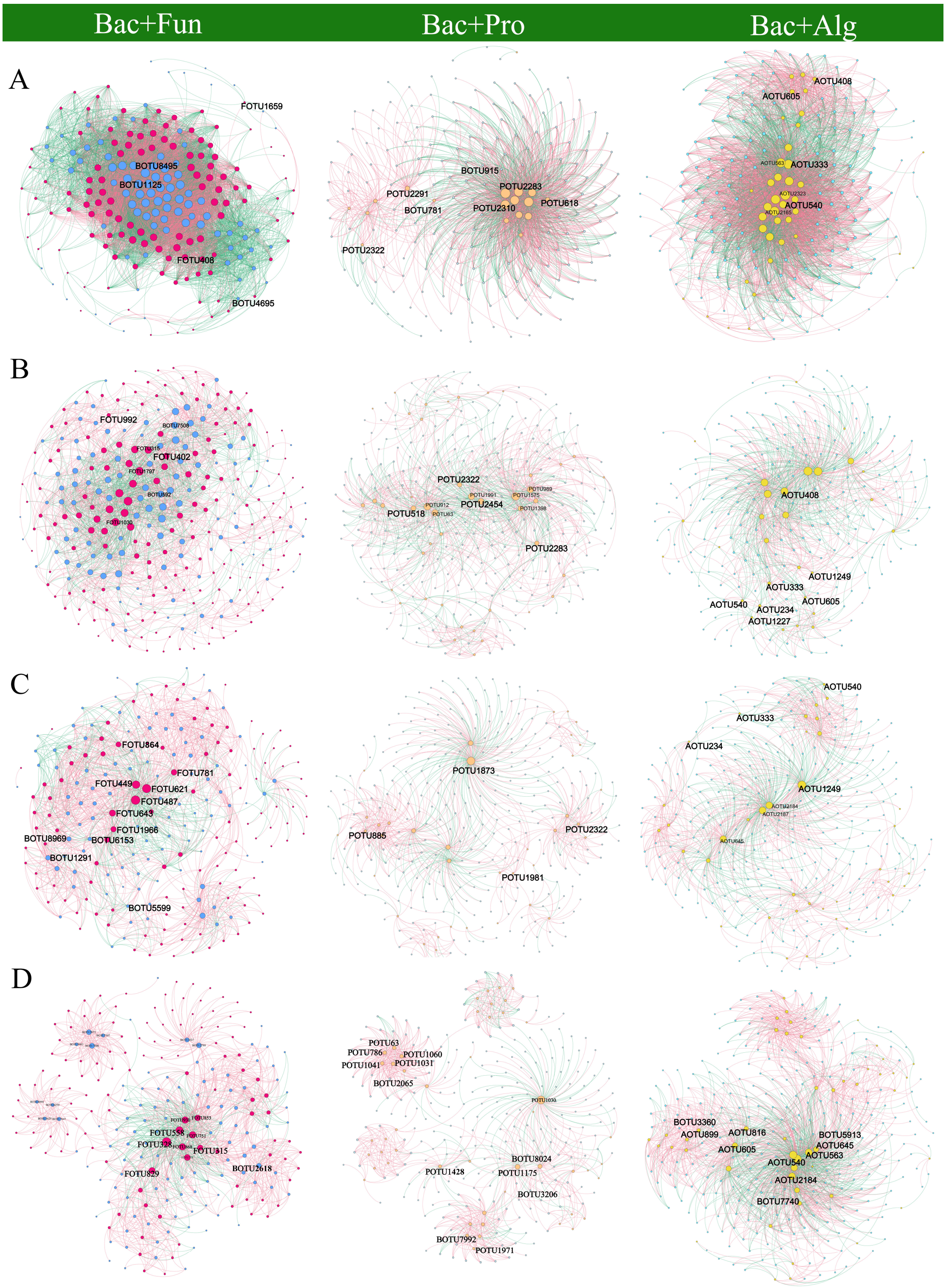

Figure S8. Bipartite co-occurrence network of bacteria and eukaryote (fungal, protozoa and alga) in overlying water (A), surface sediment (B), middle sediment (C) and bottom sediment (D). The green lines depict negative correlation and red lines depict positive correlation, with a correlation > 0.6 and *p*

< 0.05. The size of the node is proportional to its degree. Different taxa are shown in different colors in the bipartite co-occurrence network.

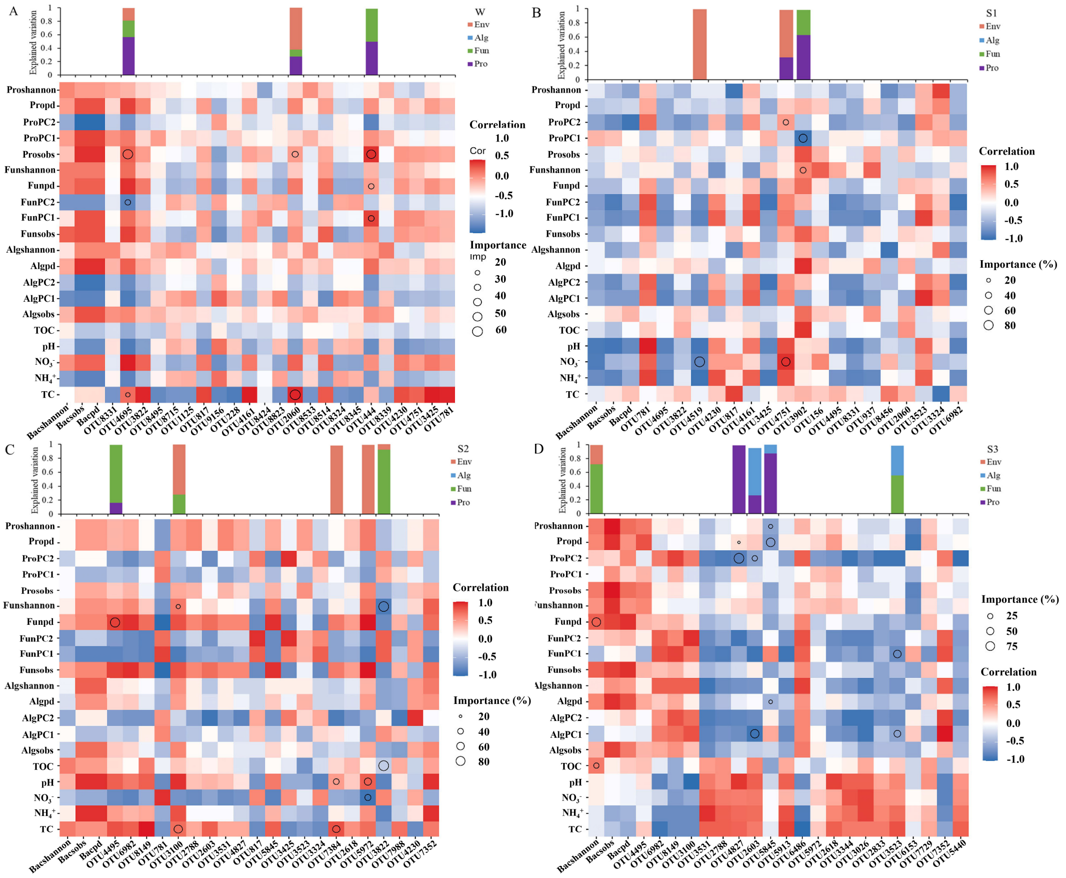

Figure S9. Contributions of eukaryote community diversity (Shannon, sobs and pd index) and physicochemical parameters to the bacterial diversity and important OTUs based on the best multiple regression model in (A) overlying water (W), (B) surface sediment (S1), (C) middle sediment (S2) and (D) bottom sediment (S3). The bar chart represents the total contribution of biotic (Pro, Fun and Alg represent diversity of protozoa, fungal and alga communities, respectively) and abiotic indicators to explain bacterial and important OTU variation (proportion of explained variability calculated via multiple regression modeling). Circle size represents the variable importance (that is, the proportion of explained variability calculated via multiple regression modeling and variance decomposition analysis). Colors represent Spearman correlations.

Table S1. Trophic status of each taxon within the protozoa communities on the genus level (> 10% of total abundance) in overlying water (W), surface sediment (S1), middle sediment (S2) and bottom sediment (S3).

Genus level	Trophic functional group	Trophic functional subgroup	S1	S2	S3	W
g__Paulinella	Phototroph	Phototroph	0.0112	0.0000	0.0002	0.0022
g__Prorocentrum	Phototroph	Phototroph	0.0053	0.0044	0.0006	0.0007
g__Asulcocephalium	Phototroph	Phototroph	0.0214	0.0053	0.0000	0.0028
g__Ceratium	Phototroph	Phototroph	0.0434	0.0158	0.0000	0.0349
g__unclassified_k__Alveolata	Parasite	Unknownparasite	0.0599	0.1085	0.0802	0.0334
g__Parvilucifera-group_X	Parasite	Unknownparasite	0.0000	0.0000	0.0075	0.2388
g__unclassified_f__Actinocephalidae	Parasite	Invertebrateparasite	0.0519	0.0073	0.0000	0.0018
g__Spongospora	Parasite	Plantparasite	0.0108	0.0031	0.0171	0.0003
g__Polymyxa-lineage_X	Parasite	Plantparasite	0.0038	0.0004	0.0167	0.0003
g__Polymyxa	Parasite	Plantparasite	0.0036	0.0000	0.0165	0.0001
g__Leidyana1	Parasite	Invertebrateparasite	0.0015	0.0031	0.0103	0.0001
g__Plasmodiophorida_XX	Parasite	Unknownparasite	0.0041	0.0024	0.0073	0.0000
g__Labyrinthulaceae_X	Consumer	Saprotroph	0.0046	0.0055	0.0126	0.0015
g__Filamoeba	Consumer	Omnivore	0.0106	0.0003	0.0043	0.0001
g__Eocercomonas	Consumer	Omnivore	0.0050	0.0034	0.0064	0.0001
g__Strobilidiidae_J_X	Consumer	Omnivore	0.0001	0.0000	0.0065	0.0954
g__Strombidiida_A_XX	Consumer	Omnivore	0.0167	0.0000	0.0000	0.0546
g__Rhogostoma-lineage_X	Consumer	Omnivore	0.0112	0.0040	0.0421	0.0043
g__unclassified_f__Rhogostoma-lineage	Consumer	Omnivore	0.0041	0.0074	0.0018	0.0002
g__Telonemia-Group-2_X	Consumer	Omnivore	0.0072	0.0043	0.0015	0.0006
g__Colpoda	Consumer	Omnivore	0.0000	0.0117	0.0011	0.0001
g__unclassified_c__Spirotrichea	Consumer	Omnivore	0.0000	0.0128	0.0000	0.0000
g__Halteria	Consumer	Omnivore	0.0044	0.0049	0.0000	0.0015
g__Protaspa-lineage_X	Consumer	Eukaryvore	0.0214	0.0152	0.0005	0.2180
g__Leptophryidae_X	Consumer	Eukaryvore	0.0333	0.0317	0.0062	0.0084
g__Aphamonas	Consumer	Eukaryvore	0.0132	0.0226	0.0142	0.0009
g__Novel-clade-2_X	Consumer	Eukaryvore	0.0009	0.0000	0.0006	0.0485
g__Penardia-lineage_X	Consumer	Eukaryvore	0.0282	0.0120	0.0000	0.0044
g__unclassified_f__Leptophryidae	Consumer	Eukaryvore	0.0070	0.0185	0.0114	0.0012
g__Litostomatea_XXX	Consumer	Eukaryvore	0.0300	0.0040	0.0000	0.0012
g__unclassified_o__Vampyrellida	Consumer	Eukaryvore	0.0092	0.0166	0.0012	0.0012
g__sm27-lineage_X	Consumer	Eukaryvore	0.0050	0.0007	0.0057	0.0013
g__unclassified_f__Sandonidae	Consumer	Bacterivore	0.0264	0.0566	0.1070	0.0013

Genus level	Trophic functional group	Trophic functional subgroup	S1	S2	S3	W
g__Allantion	Consumer	Bacterivore	0.0031	0.0833	0.0000	0.0001
g__Sandonidae_X	Consumer	Bacterivore	0.0158	0.0124	0.0195	0.0005
g__Nolandellidae_X	Consumer	Bacterivore	0.0069	0.0067	0.0211	0.0002
g__Sphaeroeca	Consumer	Bacterivore	0.0131	0.0214	0.0000	0.0003
g__Neoheteromita	Consumer	Bacterivore	0.0112	0.0115	0.0086	0.0002
g__Halteriidae_X	Consumer	Bacterivore	0.0017	0.0007	0.0001	0.0283
g__Allapsa	Consumer	Bacterivore	0.0001	0.0000	0.0190	0.0000
g__unclassified_o__Glissomonadida	Consumer	Bacterivore	0.0025	0.0027	0.0115	0.0011
g__Sandona	Consumer	Bacterivore	0.0018	0.0137	0.0015	0.0003
g__Group-Te	Consumer	Bacterivore	0.0000	0.0000	0.0126	0.0000
g__Paracercomonas	Consumer	Bacterivore	0.0105	0.0102	0.0150	0.0003
g__Glissomonadida_XX	Consumer	Bacterivore	0.0007	0.0135	0.0163	0.0000
g__Cercomonas	Consumer	Bacterivore	0.0025	0.0092	0.0007	0.0004
g__Urotricha	Consumer	Bacterivore	0.0038	0.0021	0.0000	0.0098
g__Vorticella	Consumer	Bacterivore	0.0226	0.0085	0.0060	0.0480
g__unclassified_p__Cercozoa	Unknown	Unknownnutrition	0.0678	0.1060	0.0836	0.0092
g__unclassified_c__Dinophyceae	Unknown	Unknownnutrition	0.0562	0.0577	0.0444	0.0115
g__Fibrophrys	Unknown	Unknownnutrition	0.0133	0.0137	0.0144	0.0035
g__NC12B-lineage_X	Unknown	Unknownnutrition	0.0044	0.0116	0.0284	0.0003
g__unclassified_c__Filosa-Imbricatea	Unknown	Unknownnutrition	0.0000	0.0002	0.0407	0.0004
g__Peridiniopsis	Unknown	Unknownnutrition	0.0237	0.0052	0.0000	0.0097
g__Novel-clade-10_XX	Unknown	Unknownnutrition	0.0011	0.0021	0.0153	0.0046
g__unclassified_c__Filosa-Sarcomonadea	Unknown	Unknownnutrition	0.0034	0.0032	0.0076	0.0007
g__Labyrinthula	Unknown	Unknownnutrition	0.0016	0.0110	0.0017	0.0004
g__unclassified_f__Amphifilaceae	Unknown	Unknownnutrition	0.0057	0.0008	0.0075	0.0004
g__Parvodinium	Unknown	Unknownnutrition	0.0000	0.0136	0.0000	0.0000
g__unclassified_c__Oligohymenophorea	Unknown	Unknownnutrition	0.0015	0.0073	0.0000	0.0020
g__Cercozoa_XXXX	Unknown	Unknownnutrition	0.0016	0.0007	0.0063	0.0015
g__unclassified_f__Ophryoglenida	Unknown	Unknownnutrition	0.0000	0.0260	0.0031	0.0000
g__Pseudodendromonadales_XX	Unknown	Unknownnutrition	0.0090	0.0028	0.0115	0.0030
g__Frontoniidae_1_X	Unknown	Unknownnutrition	0.0110	0.0033	0.0000	0.0076
g__Linostomella	Unknown	Unknownnutrition	0.0107	0.0000	0.0000	0.0032
g__WIM80-lineage_X	Unknown	Unknownnutrition	0.0006	0.0020	0.0094	0.0000
g__Endostelium	Unknown	Unknownnutrition	0.0005	0.0000	0.0153	0.0000
g__Spirostomum	Unknown	Unknownnutrition	0.0025	0.0059	0.0068	0.0005
g__Pseudoparamoeba	Unknown	Unknownnutrition	0.0013	0.0059	0.0052	0.0002
